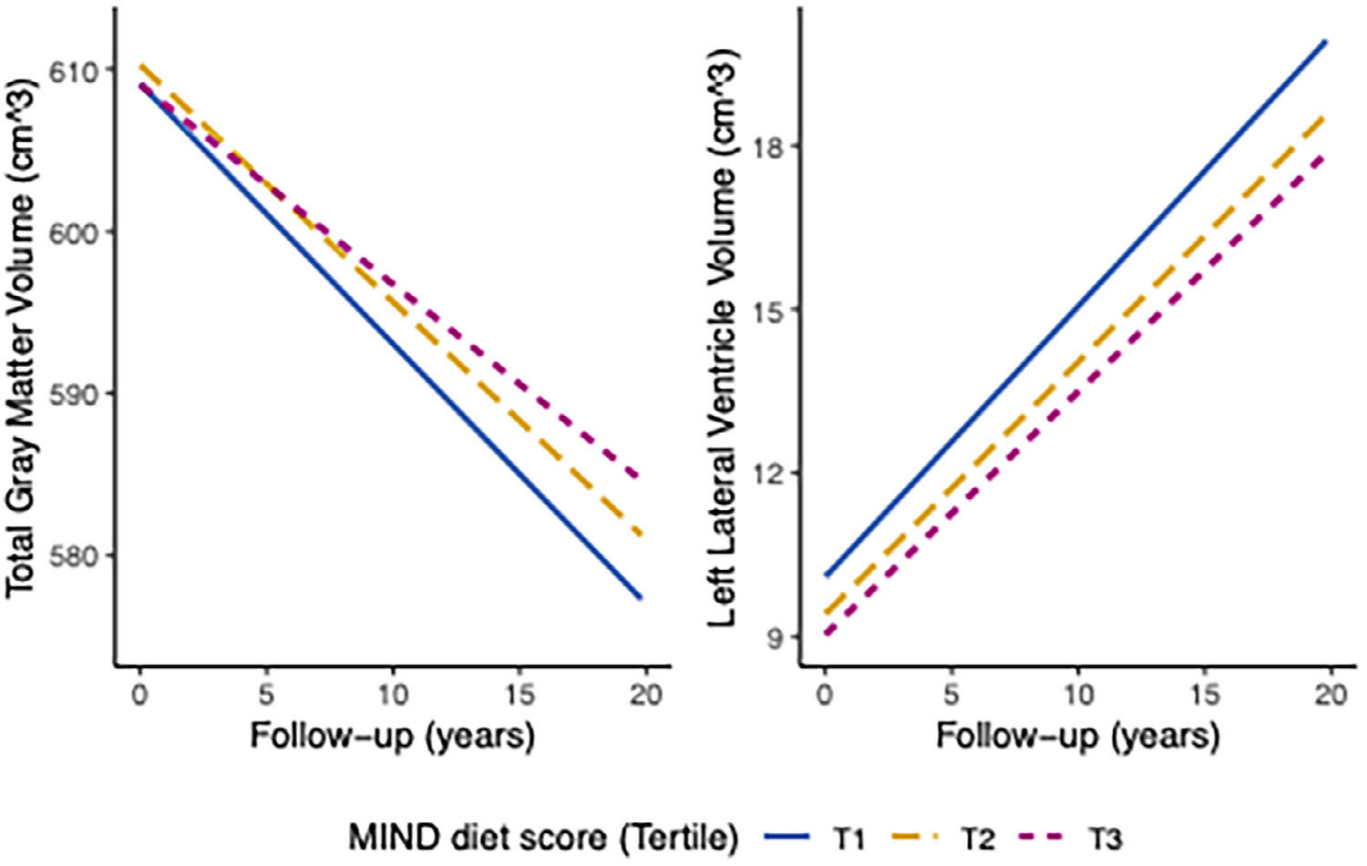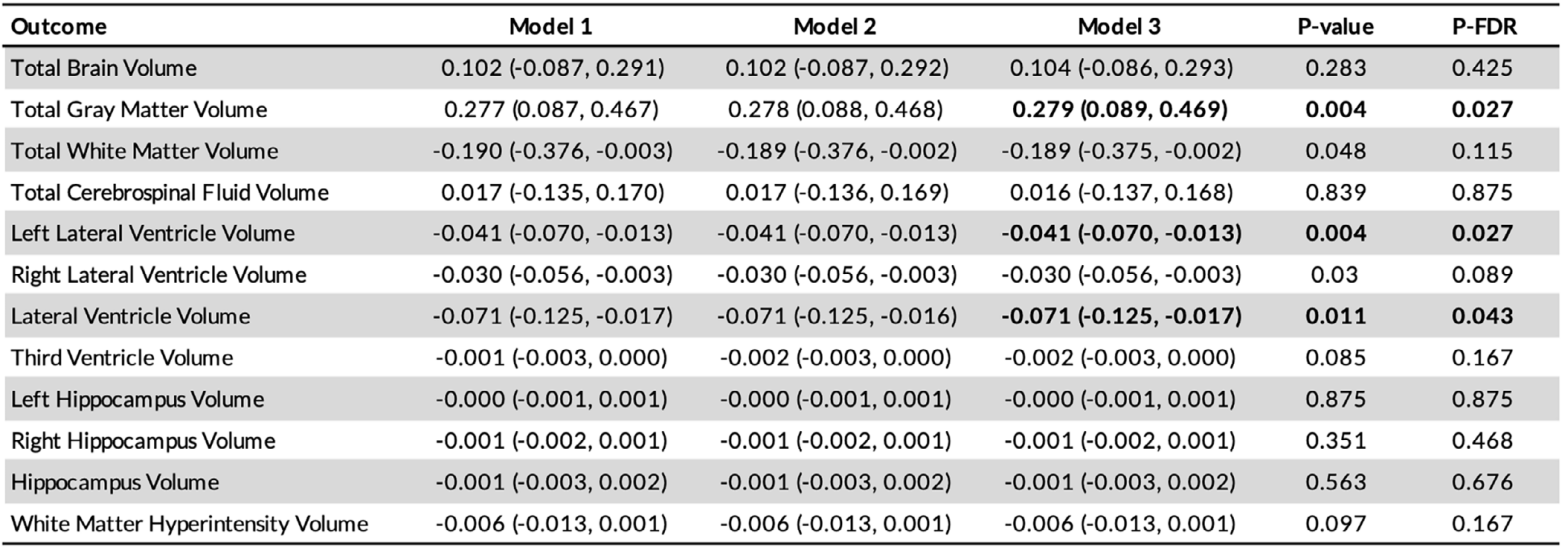# Adherence to the MIND diet and longitudinal brain structural changes over a decade: evidence from the Framingham Offspring cohort

**DOI:** 10.1002/alz70860_101611

**Published:** 2025-12-23

**Authors:** Hui Chen, Gulisiya Hailili, Xue Li, Debora Melo van Lent, Changzheng Yuan

**Affiliations:** ^1^ Zhejiang University School of Medicine, Hangzhou, Zhejiang, China; ^2^ Harvard T. H. Chan School of Public Health, Boston, MA, USA; ^3^ Zhejiang University, Hangzhou, Zhejiang, China; ^4^ Glenn Biggs Institute for Alzheimer's & Neurodegenerative Diseases, University of Texas Health San Antonio, San Antonio, TX, USA

## Abstract

**Background:**

MIND diet was favorably linked to lower risk of neurodegenerative diseases. While previous cross‐sectional studies implied its beneficial associations with brain imaging markers, its associations with long‐term brain structural changes remained unclear.

**Method:**

We included 1647 middle‐aged and older individuals from the Framingham Heart Study Offspring cohort (FOS). MIND diet score was calculated from an extensively validated FFQ in repeatedly administered at Exams 5, 6, and 7 (1991‐2001). Brain imaging markers were acquired in 1999‐2019, with a median repetition (interquartile range, IQR) of 3 (2, 3) times. We used linear mixed models to assess the associations of the MIND diet score and its components with longitudinal brain structural changes.

**Result:**

During follow‐up (median: 12.3, IQR: 6.8‐13.7 years), higher MIND diet score was associated with slower decline in total grey matter volume. Each 3‐unit increment was associated with 0.279 cm^3^/year (95%CI: 0.089, 0.469) cm^3^/year slower decline in total gray matter volume, which corresponded to 20.1% of the time effect. Higher MIND diet score was also associated with slower increment in lateral ventricle volume (‐0.071 cm^3^/year, 95%CI: ‐0.125, ‐0.017), especially left ventricle volume (‐0.041 cm^3^/year, 95%CI: ‐0.070, ‐0.013). The estimates corresponded to 8.0% and 8.8% of the time effect, respectively.

**Conclusion:**

In a community‐based prospective study, greater adherence to the MIND diet was related to slower brain atrophy for up to two decades, particularly demonstrated by slower grey matter loss and ventricle expansion. These findings supported the potential brain health benefits of the MIND diet.